# Risk factors associated with visiting or not visiting the accident & emergency department after a fall

**DOI:** 10.1186/1472-6963-13-286

**Published:** 2013-07-26

**Authors:** Alice C Scheffer, Pieter Boele van Hensbroek, Nynke van Dijk, Jan S K Luitse, Johannes C Goslings, René H Luigies, Sophia E de Rooij

**Affiliations:** 1 Department of Internal Medicine, section of Geriatric Medicine, Academic Medical Center, University of Amsterdam, Amsterdam, The Netherlands; 2 Trauma Unit, Department of Surgery, Academic Medical Center, University of Amsterdam, Amsterdam, The Netherlands; 3 Department of General Practice/Family Medicine, Academic Medical Center, University of Amsterdam, Amsterdam, The Netherlands; 4 Department of Emergency Medicine, Academic Medical Center, University of Amsterdam, Amsterdam, The Netherlands; 5 Independent Consultant, Rijswijk, the Netherlands

**Keywords:** Older persons, Falls, Risk factors, A&E Department, Mobile fall prevention unit

## Abstract

**Background:**

Little is known about the prevalence of modifiable risk factors of falling in elderly persons with a fall-history who do not visit the Accident and Emergency (A&E) Department after one or more falls. The objective of this study was to determine the prevalence of modifiable risk factors in a population that visited the A&E Department after a fall (A&E group) and in a community-dwelling population of elderly individuals with a fall history who did not visit the A&E Department after a fall (non-A&E group).

**Methods:**

Two cohorts were included in this study. The first cohort included 547 individuals 65 years and older who were visited at home by a mobile fall prevention team. The participants in this cohort had fall histories but did not visit the A&E Department after a previous fall. These participants were age- and gender-matched to persons who visited the A&E Department for care after a fall. All participants were asked to complete the CAREFALL Triage Instrument.

**Results:**

The mean number of modifiable risk factors in patients who did not visit the A&E Department was 2.9, compared to 3.8 in the group that visited the A&E Department (*p*<0.01). All risk factors were present in both groups but were more prevalent in the A&E group, except for the risk factors of balance and mobility (equally prevalent in both groups) and orthostatic hypotension (less prevalent in the A&E group). The risk factors of polypharmacy, absence of orthostatic hypotension, fear of falling, impaired vision, mood and high risk of osteoporosis were all independently associated with visiting the A&E Department.

**Conclusion:**

All modifiable risk factors for falling were found to be shared between community-dwelling elderly individuals with a fall history who visited the A&E Department and those who did not visit the Department, although the prevalence of these factors was somewhat lower in the A&E group. Preventive strategies aimed both at patients presenting to the A&E Department after a fall and those not presenting after a fall could perhaps reduce the number of recurrent falls, the occurrence of injury and the frequency of visits to the A&E Department.

## Background

Falls are a major health problem among older adults because they are frequent and may have severe consequences [1-4]. Approximately 30% of the community-dwelling persons aged 65 years and older fall at least once a year, and approximately 15% fall two or more times per year [1,5-7]. In the Netherlands, 2.6 million inhabitants are aged 65 years or older. Every year, almost 100,000 older persons are treated in hospital emergency rooms for fall-related injuries [8,9]. The consequences of falls, such as injury and disability, are a major threat to the independence and well-being of these individuals [1,10,11]. Fall-related injuries are the third leading cause of years lived with disability, according to the World Health Organization’s report ‘Global burden of disease’ [12]. In addition, falls can have considerable psychological consequences, leading to a fear of falling, depression and social isolation [13]. In older persons, a high incidence of falls is associated with a high susceptibility to injury. This susceptibility is based on the prevalence of co-morbid disease and age-related physiological deterioration and could cause serious consequences to result from a mild fall [14]. In addition, older persons who have sustained a fall are at risk of falling again and of osteoporotic fractures. Because of these consequences, both primary and secondary prevention of falls is crucial. Over the last several decades, many studies have been published regarding the prevention of secondary falls and fractures, and contradictory results have been found [15]. This disparity may have been due to differences in the populations and strategies used in these studies, and other triage strategies might therefore be useful.

Programs for the identification of modifiable risk factors and secondary prevention of falls are most often aimed at those patients presenting to the Accident & Emergency (A&E) Department after a fall [16-18]. However, little is known about the prevalence of modifiable risk factors and their association with falling in older persons with a fall history who do not visit the A&E Department after one or more falls. In this population, secondary fall prevention and primary fracture prevention should be conducted to prevent falls and their harmful sequelae [1]. Preventive activities adapted and tailored to each individual might be advisable.

The first objective of this study was to compare the prevalence of modifiable risk factors for falling in two groups of older persons with a fall history. The first group consisted of persons who did not come to the A&E Department after a fall (A&E group) and the second group included those who did visit the Department after a fall (non-A&E group). The second objective was to find modifiable risk factors related to a higher likelihood of presenting to the A&E Department after a fall.

## Methods

### Patient population

For this observational cohort study, two groups of subjects aged 65 years or older were included. The individuals in the first group were invited by mail to participate in a touring, mobile fall prevention and intervention program between July 1, 2007 and March 5, 2009. This program consisted of a mobile van, which was equipped as a small mobile diagnostic centre and was taken to visit the participants at their home addresses. If participants were interested in the program, they received a visit from a fall prevention team, during which time home care nurses assessed the modifiable risk factors for falling. Within this program, the participants were presented with an intervention program based on their identified modifiable risk factors for recurrent falling. This initiative was supported by an unrestricted grant, which was part of an innovative care project of a large, nationwide active health insurance company in the Netherlands. Individuals who had never sustained a fall were excluded from this study.

The second group consisted of patients who presented to the A&E Department of the Academic Medical Center (AMC), a university teaching hospital in Amsterdam, after a fall. The patients were selected between February 1, 2004 and July, 31, 2010. The exclusion criteria for the second group included cognitive impairment, admittance to the ICU or Department of Neurology, language problems, death within 24 hours after the fall, living in a nursing home, falls resulting from external violence or having been sent the Carefall Triage Instrument (CTI) aftert a previous visit to the A&E Department.

### The CAREFALL triage instrument (CTI)

The CTI consists of 44 questions to determine patient characteristics (age, gender, social status, living arrangements, physical activity and self-reported health), characteristics and possible cause(s) of the fall (accidental fall, fainting, or otherwise), and modifiable risk factors for falling. This self-reported fall history questionnaire was designed to identify modifiable risk factors for falling in older persons [16,18] and has proven to be reliable and valid in assessing the potential modifiable risk factors in older patients presenting to the A&E Department after a fall [16]. The CTI was also used to collect socio-demographic data.

### Procedure

When the participants were visited by the mobile fall prevention unit, they were asked to complete the CTI, in which the number of falls was specified, to allow measurement of the modifiable risk factors. When required, assistance was offered by a nurse. For the second study group, the charts of patients visiting the A&E Department were reviewed on a daily basis to identify eligible patients. All eligible patients received the CTI and a letter explaining the purpose of the study by mail, together with a stamped envelope, within one week after presentation at the A&E Department. Nonrespondents were contacted by telephone two weeks after receiving the CTI by mail and were asked to complete the questionnaire. Risk factors that could be improved or removed through intervention were defined as modifiable risk factors [18]. Based on the results of the CTI, the following modifiable risk factors were assessed: polypharmacy, complaints of orthostatic hypotension, balance and mobility disorders, fear of falling (FOF), impaired vision, urinary incontinence, presence of mood disorder symptoms and a high risk of osteoporosis [5,6,18]. Definitions of the modifiable risk factors are listed in Table 1. To obtain information regarding the location of the falls, the participants were asked if the reported falls had occurred at home or elsewhere. We aimed to match each participant from the mobile fall prevention unit group with an individual from the group who had visited the A&E Department after a fall. Participants from the two groups were matched by year of birth (up to 1 year older or younger) and gender. The matching procedure was performed according to the STROBE guideline for observational cohort studies [19]. The recruitment procedures were conducted in accordance with the Dutch Medical Research Involving Human Subjects Act and the World Medical Association’s Declaration of Helsinki. This study was part of a larger, on-going study and was approved by the Medical Ethics Committee of the Academic Medical Center, Amsterdam, the Netherlands. All participants gave informed consent for their data to be used anonymously.

**Table 1:** The definitions of the eight modifiable risk factors

***Modifiable risk factor***	***Definition***
Polypharmacy	● Use of three or more medications, independent of their types and/or
	● Use of sedative, psychoactive, anti-hypertensive, or diuretic medications
Orthostatic hypotension	One or more of the nine questions concerning orthostatic hypotension were answered positively on the CTI
Balance and mobility	● Difficulties in walking and/or
	● Use of an aid for walking and/or
	● A lack of balance and/or
	● Pain in the feet or legs and/or
	● Reduced feeling in the feet or legs and/or
	● Reduced strength in one or both feet and/or
	● Stiffness of the joints
Fear of falling	A score of 5 or more on the scale from 1 (no fear of falling) to 10 (a very large fear of falling) on the question: “are you afraid to fall?”
Impaired vision	● Unable to read the newspaper, even with magnifying glasses or a loupe, and/or
	● Substantially reduced eyesight in the past 6 months
Urinary incontinence	● Daily problems with urinary incontinence and/or
	● Need to get out of bed twice or more per night to visit the toilet
Mood	● Feeling down or depressed and/or
	● Loss of interest
	Both within the last month
High risk of osteoporosis	Patients with a fracture after the age of 50 and/or a fracture of a vertebra and/or positive for two of the three following factors:
	● Mother suffered hip fracture
	● Low body weight (men <67 kg, women < 60 kg)
	● Severe immobility

### Statistical analyses

The data were analysed using SPSS-PC software version 18.0 (SPSS inc. Chicago, Illinois). The baseline data are summarized using standard descriptive statistics: as percentages for categorical data, as means and standard deviations (SD) for normally distributed numerical data, and as medians and ranges for non-normally distributed numerical data, based on visual inspection of the histogram. Differences in the scores of normally distributed continuous variables were tested with Student’s t-test, and differences in the scores of non-normally distributed continuous variables were tested with a Mann–Whitney U test. The Chi-square test was used to analyse the differences in dichotomous variables between the two study groups. A risk factor was defined as missing for a participant if more than 50% of the CTI items constituting that risk factor were not completed [18]. The total number of risk factors for each patient was calculated by the sum score of the individual modifiable risk factors. To identify modifiable risk factors independently associated with visiting the A&E Department after a fall, we performed a logistic regression analysis. We started with a univariate logistic regression analysis. Modifiable risk factors known to be associated with falling, as described above, were included in the regression analysis. All variables with a *p*-value of ≤ 0.05 in the univariate logistic regression analysis were included in the multivariate analysis. A backward selection procedure was used, and a *p*-value of ≤0.05 was considered statistically significant.

## Results

**Figure 1:**
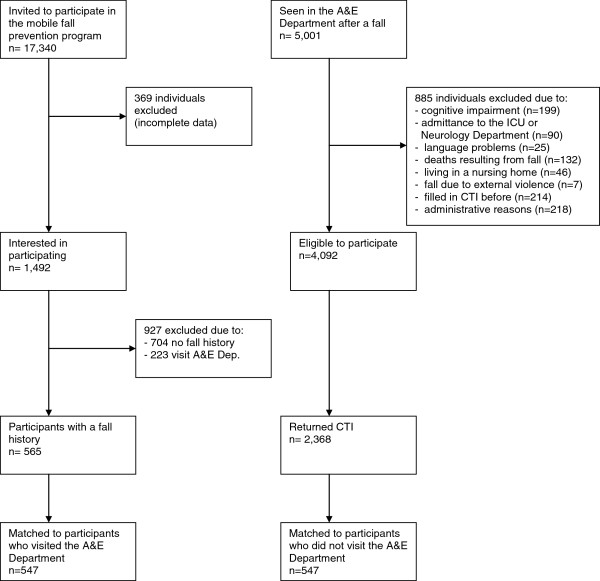
Flow chart of participants.

From the 17,340 persons invited to participate in the mobile prevention program, 1,861 older persons replied that they were interested in an assessment by the mobile team. We excluded 369 potential participants for reasons of missing or incomplete data, due to software or administrative reasons. Of the 1,492 eligible participants, 704 did not have a fall history and were therefore excluded. We also excluded 223 participants who indicated that they had visited an A&E Department after a fall within the last 12 months. This left 565 participants with a fall history who had not visited an A&E Department after sustaining a fall. During the inclusion period, 5,001 patients visited the A&E Department after a fall. Eight hundred and eighty-five patients (17.7%) of these patients were excluded, 199 patients because of severe cognitive impairment, 90 patients because of admittance to the ICU or Department of Neurology after presentation at the A&E Department, 25 patients because they were not able to speak or understand Dutch, 132 patients because of death resulting from the fall, 46 patients because of living in a nursing home, seven patients because the fall resulted from external violence, 214 patients because they were sent the CTI at an earlier visit to the A&E Department and 218 patients for administrative reasons. From the resulting 4,092 patients, 2,638 (64.4%) participants returned the CTI (Figure 1).

**Figure 2:**
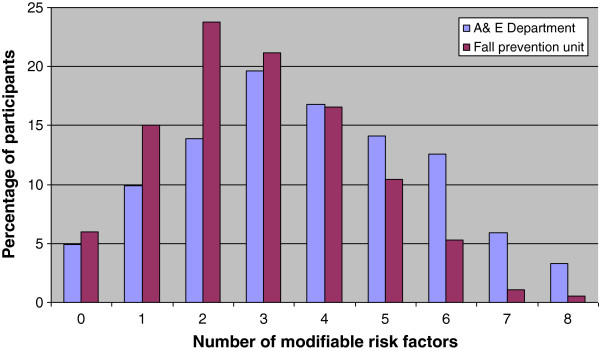
Histogram of the number of modifiable risk factors for participants from the A&E Department and from the mobile fall prevention unit (p<.01).

Matched individuals from the A&E Department cohort could be found for 547 of the 565 individuals in the mobile fall prevention cohort. Table 2 presents the baseline characteristics of the two study populations. The number of participants with more than one fall in the last 12 months was higher in the A&E group than in the non-A&E group (*p*<0.01). All modifiable risk factors were present in both study groups, but they were more prevalent in the participants who had visited the A&E Department than in the participants in the non-A&E group, except for the risk factors of balance and mobility (equal prevalence between the groups) and orthostatic hypotension (lower in the A&E Department group) (Table 3). The respondents from the A&E group had a mean of 3.8 (SD 1.8) modifiable risk factors, compared to a mean of 2.9 (SD 1.6) for the respondents from the cohort non-A&E group (*p*<0.01). Figure 2 shows the distribution of the number of modifiable risk factors for the two study groups. In the A&E group, 42.1% of the participants indicated that they fell only in their own home, while 39.5% of the participants fell only in places other than home, and 18.4% fell both at home and elsewhere. In the non-A&E group, 28.4% of the participants fell only at home, 52.1% fell only in places other than home, and 19.5% fell both at home and elsewhere (*p*<0.01).

**Table 2:** General characteristics of participants and p-values for the differences between the groups

	**Accident & emergency department cohort**	**Not visiting the A&E department cohort**	*p*-value^*^
	**(n=547)**	**(n=547)**	
Demographic			
Age in years, mean (sd)	79.1 (6.7)	79.1 (6.7)	0.98
Female (%)	69.8	69.8	1.00
Social status (%)			
Married/living together	45.5	46.3	
Widowed/divorced	41.1	43.9	
Single	13.4	9.8	
			0.05
Living arrangements (%)			
Living independently without help	49.0	56.7	
Living independently with help	43.3	40.5	
Senior residence	7.7	2.8	
			< 0.01
Physical activity (%)			
Daily	43.6	64.2	
Several times a week	13.8	14.4	
Once a week	8.8	5.9	
Once a month	0.6	0.8	
(almost) never	30.6	14.8	
Unknown	2.6	---	
			< 0.01
Going outside			
Daily	76.8	88.8	
Once a week	13.3	8.6	
Once a month	1.0	0.9	
Never	8.9	1.7	
			< 0.01
Self-reported health-related issues (%)			
Comorbid conditions			
Diabetes mellitus	16.6	14.1	< 0.01
Stroke/cerebral infarction	11.5	6.9	< 0.01
Visual impairment	28.9	21.9	< 0.01
Cancer	15.0	16.8	< 0.01
Hypertension	39.3	45.9	< 0.01
Heart failure	12.4	10.4	< 0.01
Thyroid disease	9.1	10.4	< 0.01
Other	68.6	70.7	0.25
Number of medications, median (quartiles)	3 (2–5.5)	1 (0–2)	< 0.01
Sleep medication (%)	5.4	7.3	0.23
Sedative and psychoactive (%)	9.8	4.9	< 0.01
Antidiuretic (%)	30.1	19.2	< 0.01
Antihypertensive (%)	39.9	32.4	0.02
Use of alcohol (%)	45.0	59.8	< 0.01
Smoking (%)	15.4	8.4	< 0.01
Falls and mobility (%)			
More than one fall in the last 12 months	57.3	45.3	< 0.01
Difficulty walking	42.9	42.0	0.77
Use of walking aid	38.5	28.0	< 0.01
Psychological parameters			
VAS-Fear of Falling, median (quartiles)	3 (1–7)	3 (1–4)	0.03

* *p* < 0.05.

**Table 3:** **Prevalence of modifiable risk factors in the A**&**E department group and in the non-A**&**E group**

**Modifiable risk factor (%)**	**A&E group**	**Non-A&E group**	***p*-value**
	**(n=547)**	**(n=547)**	
Polypharmacy	77.2	41.1	< 0.01
Orthostatic hypotension	37.6	48.3	< 0.01
Balance and mobility	69.1	66.0	.28
Fear of falling	41.1	17.8	< 0.01
Impaired vision	33.1	16.5	< 0.01
Urinary incontinence	67.2	52.7	< 0.01
Mood	31.9	15.9	< 0.01
High risk of osteoporosis	63.7	41.7	< 0.01
Number of modifiable risk factors, mean (sd)	3.8 (2.0)	2.9 (1.7)	< 0.01

The results of the univariate analysis are shown in Table 4. All selected variables, except for balance and mobility disorders, were associated with visiting the A&E Department after a fall. Table 4 also represents the outcomes of the multivariate logistic regression analyses. The risk factors of polypharmacy, absence of orthostatic hypotension, FOF, impaired vision, presence of mood disorder symptoms and high risk of osteoporosis proved to be independently related to a visit to the A&E Department.

**Table 4:** **Univariate and multivariate analyses for modifiable risk factors associated with visits to the A**&**E department**

	**Univariate**			**Multivariate**		
**Risk factors (%)**	**OR**	**95% CI**	*p*-value^*^	**OR**	**95% CI**	***p*-value**
Orthostatic hypotension	0.64	0.49 to 0.84	< 0.01	0.26	0.18 to 0.39	< 0.01
Balance and mobility	1.15	0.89 to 1.49	0.28			
Urinary incontinence	1.83	1.42 to 2.36	< 0.01			0.35
High risk of osteoporosis	2.46	1.92 to 3.16	< 0.01	1.98	1.41 to 2.79	< 0.01
Mood	2.47	1.84 to 3.34	< 0.01	2.38	1.53 to 3.70	< 0.01
Impaired vision	2.52	1.88 to 3.37	< 0.01	1.92	1.26 to 2.93	< 0.01
Fear of Falling	3.24	2.45 to 4.28	< 0.01	3.47	2.31 to 5.24	< 0.01
Polypharmacy	4.86	3.67 to 6.44	< 0.01	4.00	2.77 to 5.77	< 0.01

## Discussion

This study shows that although the number of modifiable risk factors present in patients who visited the A&E Department was significantly higher, the non-A&E group still had a mean of 2.9 modifiable risk factors. Additionally, five of eight modifiable risk factors were present in more than 40% of the individuals who did not visit the A&E Department.

All modifiable risk factors, except disturbances in balance and mobility and complaints of orthostatic hypotension, were more prevalent in older persons visiting the A&E Department after sustaining a fall than in those with a fall history who did not visit the A&E Department.

There were fewer recurrent fallers in the mobile fall prevention group than in the A&E group. Despite this, 45.3% of the individuals in the non-A&E group had fallen more than once in the last 12 months, compared to 57.3% in the A&E Department group. Our findings regarding the risk factors associated with presenting to the A&E Department are similar to those obtained in previous studies [17,18,20,21], which demonstrated that balance and gait abnormalities, visual impairment, peripheral neuropathy, polypharmacy, depression and advanced age are more prevalent in older patients from the A&E group than participants from the non-A&E group. Stel et al. [22] studied treatable risk factors in community-dwelling older persons and found an association between balance and mobility parameters, which could easily be measured and modified, and recurrent falling.

In our study, however, we focused on *modifiable* risk factors for falling, and therefore, our results should be useful for developing intervention studies targeted at reducing these risk factors among at-risk individuals. Because modifiable risk factors can be improved or removed by intervention [18], efforts aimed at primary prevention of fall injuries and secondary prevention of recurrent falls should focus on modifiable risk factors.

We found a higher prevalence of the modifiable risk factor ‘orthostatic hypotension’ in the non-A&E group than in the A&E group. This result may be explained by the observation participants from the non-A&E group were more active and experienced orthostatic hypotension more often than individuals in the A&E group [23]. Although the complaints of orthostatic hypotension may have been provoked by an active lifestyle, there is precedence for such cases. A study by Gangavati et al. (2011) demonstrated that systolic orthostatic hypotension, in combination with uncontrolled hypertension, increases the risk of falling in community-dwelling persons [24]. Another study has shown that the prevalence of orthostatic hypotension in adults with controlled hypertension is lower than that in adults with uncontrolled hypertension [25]. Although information regarding the effectiveness of hypertension treatment is lacking, we found a higher prevalence of hypertension in the individuals from the non-A&E group compared to participants who did visit the A&E Department. This might exemplify a higher prevalence of complaints of orthostatic hypotension in this study group. Participants from the A&E group fell did fall more at home than participants from the mobile non-A&E group. The latter reported more frequent physical activity and went outside more often than participants from the A&E group. In our study, we made a distinction between falls that occurred at home and those that occurred elsewhere. However, it was still unclear if these falls occurred indoors or outdoors. For example, a fall that occurred outside the home could mean a fall in the streets or a fall inside another house. Although the distinction between indoor and outdoor falls in our study is less clear than in earlier studies, our findings are consistent with those of previous reports in that indoor falls tend to occur more often in vulnerable people with compromised health, who presumably also have a higher rate of visiting the A&E Department after a fall, while outdoor falls tend to occur more frequently in active people [26-29]. The fact that the participants in the A&E group used an average of three medications may be an indication of compromised health.

The current study has some limitations. Information regarding falls and the circumstances of falls was based on a self-administered questionnaire completed by the participants and/or their caregivers. Therefore, some inaccuracy is undoubtedly present. This, in combination with the fact that the questionnaire was completed some time after the episode, could have led to recollection bias of the fall and its circumstances, especially in the age group studied [16]. Another limitation is the lack of information concerning the cognitive functioning of the included participants. Cognitively impaired persons may be at particular risk of falling and of serious sequelae when they fall [14]. Patients with severe cognitive decline were excluded from the A&E group. However, these individuals were only excluded when information on the A&E chart indicated that the patient had severe cognitive impairment. As patients from the group that did not visit the A&E Department were all living independently and had to volunteer actively to participate, it was assumed that the presence of cognitive impairment was not high. Another potential limitation was the exclusion of patients who were admitted to the ICU or the Department of Neurology from the A&E Department Group. This could have led to an inclusion of fewer persons with a high number of modifiable risk factors in this study group and therefore an underestimation of the difference in the number of modifiable risk factors between the two study groups.

The results of this study have implications for both public health and clinical practice. Community-dwelling elderly persons with a fall history who did not visit the A&E Department exhibited several modifiable risk factors for falling. Although this number of risk factors was lower than that of patients visiting the A&E Department, it was still high, and here we counted only the *modifiable* risk factors. Therefore, secondary prevention of falls should also be conducted for these individuals, as they are still at high risk for recurrent falls with major injury. The early identification of members of this group and further preventative actions, including providing more information for these persons to reduce fall risk and concomitant injury, are essential.

The identification of several risk factors supports the multifactorial nature of falls and suggests that a multidimensional, rather than a single, intervention strategy may result in the greatest risk reduction in elderly individuals [14]. Many studies and guidelines have focused on multifactorial fall-risk assessment to provide interventions, often aimed at older persons with the highest risk of recurrent falling, but the use of incorrect selection criteria could negatively impact the efficacy of such interventions [15]. The effect of multiple interventions to prevent new falls is still the focus of international discussion. Falls in community-dwelling older people could be prevented through a reduction in the number of modifiable risk factors and by creating awareness of the increased fall risk associated with the use of sedatives and benzodiazepines. New studies may further clarify if early intervention can reduce the occurrence of injury and visits to the A&E Department in this group of individuals.

## Conclusion

Although all modifiable risk factors for falling were shared between community-dwelling elderly individuals with a fall history who did not visit the A&E Department after a fall and those who did visit the A&E Department after a fall, there was a slightly lower prevalence of these risk factors in the former group. Preventive strategies aimed at both individuals presenting to the A&E Department after a fall and individuals not presenting to the Department after a fall could potentially reduce the incidence of recurrent falls, the occurrence of injury and the number of visits to the A&E Department.

## Abbreviations

A&E: Accident & emergency; CTI: Carefall triage instrument; FOF: Fear of falling; ICU: Intensive care unit.

## Competing interests

The authors declare that they have no competing interests.

## Authors’ contributions

SE developed the original idea for the study. SE, PBH and AC designed the study protocol. AC and PBH executed the study and the statistical analyses. SE, ND, PBH and AC were involved in the interpretation of data. AC and PBH drafted the manuscript. SE, ND, CG, RL and JS were involved in reviewing the manuscript. All authors approved the manuscript.

## Pre-publication history

The pre-publication history for this paper can be accessed here:

http://www.biomedcentral.com/1472-6963/13/286/prepub
